# Effects of Preoperative Use of Oral Dextromethorphan on Postoperative Need for Analgesics in Patients With Knee Arthroscopy

**DOI:** 10.5812/aapm.11187

**Published:** 2013-12-26

**Authors:** Saeid Reza Entezary, Saeedeh Farshadpour, Mahmood Reza Alebouyeh, Farnad Imani, Mohammad Kazem Emami Meybodi, Habibollah Yaribeygi

**Affiliations:** 1 Department of Anesthesiology, Rasoul Akram Medical Center, Iran University of Medical Sciences, Tehran, Iran; 2 Department of Orthopedic, Faculty of Medicine, Baqiyatallah University of Medical Sciences , Tehran, Iran; 3 Department of Physiology, Faculty of Medicine, Baqiyatallah University of Medical Sciences, Tehran, Iran

**Keywords:** Pain, Postoperative Period, Analgesics, Opioid, Analgesia

## Abstract

**Background:**

Studies have shown that N-methyl-D-aspartate receptor (NMIDA) plays an essential role in postoperative pain. It seems that use of NMDA receptor antagonists such as Dextromethorphan intensifies the analgesic effects of opioids.

**Objectives:**

In this study, we evaluated the effect of preoperative administration of Dextromethorphan on postoperative pain reduction.

**Patients and Methods:**

This double blind randomized clinical trial was conducted on arthroscopic surgery candidates. Participants were randomly allocated to interventions and assigned to two groups of Dextromethorphan and placebo. In Dextromethorphan group, the patients received 1 mg/kg Dextromethorphan orally the night before the operation. Pain severity based on the visual analog scale (VAS) up to 16 hours postoperation, use of opioids, and the first request for analgesics were recorded postoperatively.

**Results:**

A total of 112 patients in the Dextromethorphan (n = 54) and placebo groups (n = 58) were evaluated. No significant difference was detected between the two groups for age, sex or ASA. The mean amount of opioid consumption was significantly lower in patients who received Dextromethorphan (10.7 ± 5.6 mg) compared to the placebo group (13.1 ± 5.6 mg), (P = 0.03). The mean time until the first opioid request in patients who received Dextromethorphan was longer than that in the placebo group (P = 0.01).

**Conclusions:**

The study results demonstrated that preemptive use of Dextromethorphan reduced postoperative pain and opioid consumption.

## 1. Background

Postoperative pain management is an important part of postoperative care and plays an important role in postsurgical ambulation and general status of patients (1-4). Also, perioperative pain can adversely affect patient’s hemodynamic status, and increase the risk of several cardiovascular complications like myocardial infarction and cerebrovascular accidents such as stroke, and etc. (1, 2). Some studies have shown different effects of various forms of a drug on pain relief (5-7). Peri- and postoperative pain can lead to central sensitization and subsequent increased perception of pain by patients. This converts acute postoperative pains to chronic pains per se (1). Some methods have been proposed to decrease pain after surgery (8). Elimination of perioperative pain by using opioids is a common method which is followed by their adverse side effects (2). To decrease these complications, other nonopioid medications can be used to relieve pain before the operation (9-13) or after it (14, 15). Peripheral tissue damage and subsequent inflammatory response cause a local change in sensitivity of peripheral receptors and increase the excitability of neurons present in the spinal cord (2, 4). Studies have demonstrated that N-methyl-D-aspartate receptors (NMDA) play a part in increasing the sensitivity of spinal receptors (15). Therefore, considering this mechanism, a preventive method namely preemptive analgesia has been used to decrease postoperative pain recently (6). Furthermore, concomitant use of NMDA receptor antagonists and opioids can enhance the analgesic effects of opioids and stop tolerance to the analgesic action of opioids.

At present, there is a possibility that preemptive analgesia with NMDA receptor antagonists decreases postoperative pain of patients. Dextromethorphan, ketamine, amantadine, and magnesium sulfate are among the well-known NMDA receptor antagonists. Dextromethorphan and its metabolites are considered as noncompetitive low affinity NMDA receptor antagonists (15). Considering the fact that Dextromethorphan is a widely available oral medication and has minimal side effects when administered in low dosage, it has been used for pain control in various studies. 

Several researchers have evaluated the analgesic effects of Dextromethorphan and its impact on peri-and postoperative need for analgesics (15). In one study it was demonstrated that administration of Dextromethorphan prior to abdominal surgery decreased the need for postoperative opioids significantly compared to the placebo group (9). Also, Dextromethorphan has been successfully used to relieve postoperative pain following mastectomy, hysterectomy, and diagnostic laparoscopy (8, 10). Considering the relevant literature, it seems that Dextromethorphan is effective in decreasing postoperative pain in patients and reduces administration of opioids. However, its preoperative use has yet to be thoroughly investigated. Further research is required to study the preoperative administration of Dextromethorphan in different groups of patients and various surgical procedures. 

## 2. Objectives

Considering the effects of Dextromethorphan on decreasing the need for opioids and subsequent reduction in their side effects, causing preemptive analgesia and less need for administration of perioperative analgesics to prevent central sensitization and reducing the incidence of chronic pain, the present study aimed to compare the effect of Dextromethorphan and placebo on reducing the need for administration of opioids and subsequent reduction in their potential side effects.

## 3. Patients and Methods

In this randomized double blind controlled clinical trial, candidates for arthroscopic knee surgery admitted to the Orthopedic Department of Rasoul-e-Akram Hospital during 2008-2009 were recruited using convenience sampling. The inclusion criteria were as follows:

- Being a candidate for arthroscopic knee surgery 

- Spinal anesthesia 

- Older than 18 years 

- ASA classes I and II

The exclusion criteria were:

- Obese patients

- Not consenting to spinal anesthesia

- Conversion of arthroscopic knee surgery to open knee surgery

- History of opioid consumption

- Use of sedatives or analgesics peri operatively

After patient selection based on the inclusion and exclusion criteria, they were assigned to the Dextromethorphan and placebo groups alternately. All patients were visited the night before the operation and signed a written informed consent. Instructions were given on how to use the VAS ruler to measure subjective complaints of pain, and the whole procedure was thoroughly explained to the subjects. In the Dextromethorphan group, patients consumed oral Dextromethorphan syrup 1 mg/kg 2-3 hours before the operation (due to its short half-life). The placebo group consumed normal saline. Also, all patients took a 10 mg Diazepam tablet. In the operating room, an IV line was taken and 500 cc of IV fluid was administered. Patients were placed in a seated position while being monitored, and Marcaine 0.5% was injected as the analgesic agent (per body weight and about 3-3.5 mL at all) into the epidural space at L4/L5 with a 25 gauge needle. Patient’s position was then quickly changed to supine and blood pressure was monitored. After completion of the operation, patients were transferred to the recovery room and then to the ward. The time of the first opioid request after the operation, and the intensity of pain at different time points were recorded.

Demographic characteristics of patients including age, sex, surgical indication, and etc. were extracted from their medical files or asked directly and recorded in data collection sheets. Severity of pain in patients was measured 4, 8, 12, 16 and 24 hours postoperatively using the visual analog scale. The patients were received analgesic drug (Morphine) every four hours or PRN when needed. The time of first opioid request after the operation was also recorded as an indicator of postoperative pain severity from the moment of entering the recovery room to patient’s first complaint of pain in minutes. Eventually, the total amount of Morphine administered within 24 hours was evaluated as an indicator of pain intensity.

The obtained data were statistically analyzed using SPSS version 13 software. Quantitative data were represented as mean and standard deviation, and qualitative data as frequency. To compare the qualitative and quantitative data between the two groups, in case of normal distribution of data, chi square test and t-test were used, respectively. If data were not normally distributed, nonparametric statistical tests were used. Also repeated measurements ANOVA was used to evaluate the effect of time and its correlation with drug (P < 0.05 was considered statistically significant). 

## 4. Results

A total of 112 patients in the two groups of Dextromethorphan (n = 54) and placebo (n = 58) were evaluated. No side effects about Dextromethorphan administration in these patients were reported. Table 1 summarizes the demographic and clinical characteristics of patients. As observed, no statistically significant difference existed between the two groups for age, gender or ASA status. 

**Table 1.** Demographic Characteristics of Patients in the Two Groups

	Dextromethorphan (n = 54)	Placebo (n = 58)	P value
**Age, mean (SD), y**	28.1 ± 8.6	30.2 ± 7.5	0.1
**Frequency of males, %**	(66.7) 36	(63.8) 37	0.7
**ASA status, %** ^**a**^			
ASA I	(70.4) 38	(77.6) 45	0.3
ASA II	(29.6) 16	(22.4) 13	0.3

^a^Abberviations: ASA, acetylsalicylic acid.

Table 2 shows the severity of pain during the first 16 hours after the operation. As observed, the mean score of pain during the first 8 hours after the operation in the Dextromethorphan group was less than that in the placebo group. During the first 12 and 16 hours postoperatively, the mean score of pain in the test group was also lower than the placebo group, but this difference was not statistically significant. According to the Repeated Measures ANOVA, the decreasing trend of pain severity in the Dextromethorphan group was significantly greater than the placebo group (P = 0.001). Time factor also played a part in this respect, and a part of pain reduction was attributed to the passing of time (P = 0.001). The correlation between premedication and time was not statistically significant (P = 0.121). 

**Table 2.** Severity of Postoperative Pain in the Two Groups

	Dextromethorphan Group(n = 54) ^a^	Placebo Group (n = 58) ^a^	P value
**In the recovery room**	4.1 ± 1.8	4.7 ± 1.2	0.05
**4 hours postoperation**	2.9 ± 0.9	3.8 ± 1.1	0.001
**8 hours postoperation**	2.7 ± 0.7	3.4 ± 0.6	0.001
**12 hours postoperation**	2.4 ± 0.6	2.5 ± 0.7	0.381
**16 hours postoperation**	0.9 ± 0.6	1.2 ± 0.7	0.09

^a^ Mean ± SD

Administration of opioids as analgesics during the first 24 hours postoperation is demonstrated in Table 3. The mean amount of opioid consumption was 10.7 ± 5.6 mg in the Dextromethorphan group, which was significantly lower than the mean rate of consumption in the placebo group (13.1 ± 6.1 mg), (P = 0.032). The mean time until the first opioid request after the operation was 3.7 ± 1.3 hours in the Dextromethorphan group, which was significantly longer than the time in the placebo group (P = 0.011) (Table 3). For occurrence of side effects due to the administration of opioids, 13 (24.1%) subjects in the Dextromethorphan and 23 (39.7%) in the placebo group experienced nausea and vomiting. Although the prevalence of these complications in the placebo group was greater than the test group, this difference was not statistically significant (P = 0.08). Incidence of itching was not significantly different between the two groups of Dextromethorphan (18.5%) and placebo (20.7%), (P = 0.773). 

**Table 3.** Severity of Pain After the Operation in the Two Groups

	Dextromethorphan (n = 54)	Placebo (n = 58)	P value
**Opioid consumption, mg, Mean ± SD**	10.7 ± 5.6^a^	13.1 ± 6.1^a^	0.03
**Time of the first analgesic postoperation, mean ± SD, hours**	3.7 ± 1.3^a^	3.1 ± 1.2^a^	0.01
**Frequency of side effects of opioids ,%**			
**Nausea and vomiting, %**	(24.1) 13	(39.7) 23	0.08
**Itching, %**	(18.5) 10	(20.7) 12	0.7

^a^Mean ± SD

## 5. Discussion

This study showed that preemptive oral Dextromethorphan can reduce the need for administration of opioids after arthroscopic knee surgery compared to the placebo. Also, it was demonstrated that the need for first analgesic was significantly later in the test group compared to the placebo group. As mentioned earlier, postoperative pain is a serious problem in surgical wards and plays a critical role in patient’s postoperative general status and their discharge time from the hospital (1, 2). In contrast to what was previously believed, assessment of pain reduction interventions by measuring pain severity at different time points cannot be a suitable indicator for their effectiveness (1) because ethically, analgesics have to be administered for patients who experience severe pains (i.e. VAS > 3). Therefore, the main key in studies conducted on control and management of postoperative pain is to evaluate the total consumption of opioids and reduced consumption in a group of patients is indicative of the effectiveness of that specific intervention (1). As mentioned earlier, the changes in pain severity at different time points were similar in the both groups, but the rate of opioid consumption was significantly lower in the test group compared to the placebo. During the recent years, Dextromethorphan has been used as an N-methyl-d-aspartate receptor channel blocker by the action of its metabolite Dextrophan (6). Recent studies have shown that NMDA receptor plays a part in increasing the central sensitization. Therefore, we can decrease postoperative pain by blocking this receptor before the operation using preemptive methods (5). Furthermore, it seems that simultaneous administration of NMDA receptor antagonists and opioids can enhance the analgesic effect of opioids and stop tolerance to analgesic effects of opioids (7). In a study in 1999, it was demonstrated that preemptive Dextromethorphan decreased pain severity and amount of Morphine administration significantly after the operation which is in accordance with our findings. Furthermore, other findings regarding this subject confirm the effectiveness of Dextromethorphan. This is in accordance with physicians’ intentions to decrease the administration of opioids. 

Although reduced consumption of opioids leads to a reduction in their potential side effects like nausea, vomiting and itching, and also in the present study, incidence of these complications was somehow lower in the test group, this difference was not statistically meaningful. 

In general, it appears that Dextromethorphan can be effective to reduce postoperative pain as a safe drug available over the counter (OTC) if administered preemptively. However, to generalize the results to other surgical procedures, further investigations are required. 

The present study results revealed that administration of Dextromethorphan can reduce consumption of opioid analgesics compared to the placebo after arthroscopic knee surgery. However, this study failed to significantly reduce the incidence of opioid side effects by preoperative consumption of Dextromethorphan in comparison to the placebo.
